# Psychiatric History and Overactive Bladder Symptom Severity in Ambulatory Urogynecological Patients

**DOI:** 10.3390/jcm10173988

**Published:** 2021-09-03

**Authors:** Artur Rogowski, Maria Krowicka-Wasyl, Ewa Chotkowska, Tomasz Kluz, Andrzej Wróbel, Dominika Berent, Paweł Mierzejewski, Halina Sienkiewicz-Jarosz, Adam Wichniak, Marcin Wojnar, Jerzy Samochowiec, Katarzyna Kilis-Pstrusinska, Przemyslaw Bienkowski

**Affiliations:** 1 Faculty of Medicine, Collegium Medicum, Cardinal Stefan Wyszynski University in Warsaw, 01-938 Warsaw, Poland; 2 Department of Obstetrics and Gynecology, Mother and Child Institute, 01-211 Warsaw, Poland; makrowicka@gmail.com (M.K.-W.); ewa.chotkowska@imid.med.pl (E.C.); 3 Department of Gynecology and Obstetrics, Institute of Medical Sciences, Medical College of Rzeszow University, 35-310 Rzeszów, Poland; jtkluz@interia.pl; 4 Second Department of Gynecology, Medical University of Lublin, Jaczewskiego 8, 20-954 Lublin, Poland; wrobelandrzej@yahoo.com; 5 Regional Psychiatric Hospital Drewnica, 05-091 Zabki, Poland; dominikaberent@poczta.fm; 6 Departments of Pharmacology, Institute of Psychiatry and Neurology, 02-957 Warsaw, Poland; pawelmierzej@yahoo.com; 7 Department of Neurology I, Institute of Psychiatry and Neurology, 02-957 Warsaw, Poland; jarosz@ipin.edu.pl; 8 Department of Psychiatry III, Institute of Psychiatry and Neurology, 02-957 Warsaw, Poland; wichniak@gmail.com; 9 Department of Psychiatry, Medical University of Warsaw, 02-091 Warsaw, Poland; marcin.wojnar@wum.edu.pl (M.W.); pbienko@yahoo.com (P.B.); 10 Department of Psychiatry, Pomeranian Medical University, 70-111 Szczecin, Poland; samoj@pum.edu.pl; 11 Department of Pediatric Nephrology, Wroclaw Medical University, 02-091 Wroclaw, Poland; katarzyna.kilis-pstrusinska@umed.wroc.pl

**Keywords:** overactive bladder, urinary incontinence, psychiatric comorbidity, psychotropic medication, urogynecology

## Abstract

Introduction and hypothesis: A link between psychiatric comorbidities and overactive bladder symptomatology has been suggested by preclinical and clinical studies. Given this, we hypothesized that a psychiatric history and current treatment with psychotropic medications could be related to the severity of overactive bladder and incontinence symptoms in patients referred to a tertiary care urogynecological center. Methods: One hundred and twenty-seven female patients diagnosed with an overactive bladder were screened for a lifetime history of psychiatric disorders and the type and number of psychotropic medications currently taken. The overall severity of overactive bladder symptoms was assessed using the Indevus Urgency Severity Scale. The severity and impact of urinary incontinence on the quality of life were quantified with the International Consultation on Incontinence Questionnaire-Urinary Incontinence Short Form. Urinary incontinence was further quantified with the aid of the Urinary Distress Inventory-6. The patients were screened for stress urinary incontinence using the Stamey Incontinence Score. Results: A psychiatric history, as well as current use of at least two psychotropic medications, was associated with increased severity of overactive bladder symptoms. A history of depression and current treatment with any selective serotonin reuptake inhibitor was associated with increased severity of stress urinary incontinence symptoms. Current treatment with other psychotropic medications, including sedative-hypnotics and drugs with anticholinergic properties was not related to the severity of overactive bladder and incontinence symptoms.

## 1. Introduction

Overactive bladder (OAB) is a symptom-based clinical diagnosis defined by the International Continence Society (ICS) in its Standardization of Terminology of Lower Urinary Tract Function [1]. It is characterized by urinary urgency with, or without, urge urinary incontinence (UUI). OAB is usually accompanied by frequent micturition and nocturia but, by definition, an absence of urinary tract infection or other underlying diseases [2,3,4].

Several factors have been implicated in OAB pathophysiology, including central and peripheral nervous system lesions and local neurotransmitter imbalance [5,6]. Urothelium, suburothelium, detrusor, and urethra have been suggested as possible anatomical sources of OAB symptoms [5]. The role of psychiatric comorbidities in urinary urgency and UUI has also been postulated. In a cross-sectional study based on an Internet survey, anxiety symptoms, but not depression, were predictive of bothersome OAB symptoms [7]. Bradley et al. [8] using computer-assisted telephone interviews assessed a longitudinal association between selected mental health conditions and OAB in women veterans. They found that an anxiety disorder, namely post-traumatic stress disorder (PTSD), but not depression, was predictive of de novo OAB symptoms. Depression, but not PTSD, was predictive of OAB remission. In a recent review paper, Tarcan et al. [9] concluded that due to their frequency, high rate, and relevance in clinical practice, screening for psychiatric comorbidities ought to be recommended for all age groups with idiopathic lower urinary tract dysfunctions. However, it is worth noting that many studies of the association between OAB and psychiatric disorders have focused on non-clinical samples approached via telephone- or Internet-based instruments and that psychotropic medications taken by study participants have been typically neglected [8,10].

Many peripheral and central neurotransmitters, such as serotonin (5-HT), ɣ-aminobutyric acid (GABA), and acetylcholine (ACh), are thought to be involved in the pathophysiology of both OAB and psychiatric disorders. These neurotransmitters are also the target for commonly used psychotropic medications [5,11,12,13,14,15,16]. Animal studies have revealed that lowering serotonin levels in the brain may be accompanied by frequent urination and detrusor overactivity [15,16]. Serotonin reduction is also thought to contribute to depression and selective serotonin reuptake inhibitors (SSRIs) are effective in depression treatment [11,12]. Likewise, GABA and GABAergic neurotransmission has been implicated in the pathomechanism of OAB [17] and several mental disorders [18]. Zolpidem, a hypnotic medication acting as a subtype-selective GABA_A_ receptor agonist, appears to increase bladder capacity and decrease urine production in rats [19], while diazepam, a benzodiazepine GABAergic drug with sedative-hypnotic properties, prolongs micturition intervals in a rat model of OAB [20].

Antagonists of muscarinic acetylcholine receptors, such as solifenacin and tolterodine, are widely prescribed to OAB patients [21]. Importantly, many psychotropic medications used in the treatment of depression, anxiety, and insomnia (e.g., amitriptyline, paroxetine, hydroxyzine, opipramol, quetiapine, olanzapine) have peripheral and central anticholinergic side effects and, in theory, at least, could modify the severity of OAB and incontinence symptoms [18,22].

The course and severity of OAB, including the presence or absence of UUI, are of primary importance for planning OAB treatment [4,23,24]. Given the above, surprisingly little is known about any possible relationship between a lifetime psychiatric history and psychotropic medications currently taken and the severity of OAB, including UUI, in ambulatory urogynecological patients. For example, to the best of our knowledge, the association between treatment with SSRIs or GABAergic medications (e.g., zolpidem, benzodiazepines) and the severity of OAB symptoms, including UUI, has never been assessed with a cross-sectional protocol. It is also largely unknown whether psychotropic medications with anticholinergic properties could reduce the severity of OAB mimicking the action of peripheral muscarinic receptor antagonists used in urogynecology [25].

The primary aim of the present study was to evaluate whether a lifetime psychiatric history and current treatment with psychotropic medications could be associated with the severity of OAB, including UUI. To address this aim, female patients referred to a tertiary care urogynecological center by their family physicians for further diagnosis and treatment of OAB were screened for a history of psychiatric disorders [26,27] and the type and number of psychotropic medications currently being taken. Notably, psychotropic medications are commonly used as a simple indicator of mental health problems in different populations of patients [28,29].

The study was cross-sectional in nature and hence our data cannot be used to infer causality.

## 2. Materials and Methods

### 2.1. Patients

The study was carried out in accordance with the Declaration of Helsinki of the World Medical Association. The study protocol was approved by the Ethics Committee for Human Studies of the Mother and Child Institute, Warsaw, Poland. All participants signed an informed consent form after study procedures had been fully explained.

Female patients aged ≥18 years, referred by their family physicians to a urogynecological ambulatory center for further diagnosis and treatment of OAB from May 2018 to December 2019, were considered potential participants. Exclusion criteria were: active urinary tract infection, urinary tract cancer, current radio- or chemotherapy, previous bladder or urethral surgery, pelvic organ prolapse stage > II, based on the Pelvic Organ Prolapse Quantification (POP-Q) system [30], diabetes, pregnancy, and lactation. Women with previous POP or stress urinary incontinence (SUI) surgery were also excluded from the study. Few patients underwent cervical surgery because of mild or moderate dysplasia, laparoscopic removal of benign ovarian cyst, and surgery of uterine myomas (ns < 10 per group).

One hundred and twenty-seven patients diagnosed with OAB, as described below [2,4], were finally included in the study.

### 2.2. OAB and Incontinence Symptoms

All conditions, methods, definitions, and units conformed to the standards recommended by the International Urogynecological Association and the ICS [31]. The patient’s evaluation followed institutional protocol, including detailed medical history, systematic physical examination, and a cough stress test (CST). A vaginal examination was performed with the patient in a semi-lithotomy position. The CST was carried out with the patient in the supine lithotomy position with the bladder comfortably filled with 200–400 mL of urine. Leakage of urine from the urethral meatus simultaneous with a cough was considered a positive test [32].

OAB was diagnosed according to the ICS definition in its Standardization of Terminology of Lower Urinary Tract Function [2,31]. It was assumed that when symptoms of both urinary urgency and frequency, with or without urinary incontinence, were present and self-reported as bothersome, the patient could be diagnosed with OAB [2,4]. The presence of OAB symptoms (urgency, frequency, and UUI) was confirmed using questions selected from the Pelvic Floor Distress Inventory as formulated by Foster et al. [33] and Rogowski et al. [34].

The self-reported severity of OAB symptoms was assessed with the aid of the Indevus Urgency Severity Scale (IUSS) [35,36]. Urinary incontinence was further quantified with the Urinary Distress Inventory-6 (UDI-6) [37,38,39]. The severity and impact of urinary incontinence on the quality of life were evaluated with the International Consultation on Incontinence Questionnaire-Urinary Incontinence Short Form (ICIQ-UI-SF) [39,40].

The patients were also interviewed about SUI symptoms using the Stamey Incontinence Score (grade 0: continent; grade 1: loss of urine with a sudden increase in abdominal pressure, such as coughing, sneezing, laughing; grade 2: leaks with lesser degrees of physical stress, i.e., walking, sitting up in bed; grade 3: urine is lost without any relation to physical activity or body position) [41]. Patients with UUI and a score of ≥1 on the Stamey test and/or showing a positive CST were considered to have mixed urinary incontinence (MUI).

### 2.3. Psychiatric History and Current Treatment with Psychotropic Medications

A brief questionnaire was designed and checked for clarity with a group of psychiatric and gynecological female patients before the start of the main study. The questionnaire was used to assess the self-reported lifetime history of psychiatric disorders [26,42] and current use of psychotropic medications [29,43]. Each patient was asked: (1) whether she had ever been treated for any psychiatric disorder in an outpatient or inpatient setting, (2) whether she had ever been under the care of a psychiatrist. Similar but separate questions were included to assess a lifetime history of depression (“Have you ever been treated for depression in an outpatient or inpatient setting?”, “Have you ever been under the care of a psychiatrist because of depression?”). In each case, an attempt was made to verify the patient’s responses with available medical files and individual lists of prescriptions.

Prescribed psychotropic medications taken in the past 30 days were identified [18,22] and counted. Subgroups of patients taking none, one or more (≥1) or two or more (≥2) psychotropic drugs were formed for further analyses. It should be remembered that some individuals may take psychotropics (e.g., sedative-hypnotics) without a specific psychiatric diagnosis and some subjects may take these medications for non-psychiatric disorders. Hence, the number of subjects with a psychiatric history and the number of subjects currently taking psychotropic medications may not be equal [28,29].

Sedative-hypnotics and antidepressants were classified according to their key pharmacodynamic characteristics as described in modern textbooks of clinical psychopharmacology [18,22]. The sedative-hypnotics were further divided as “GABAergic” (including non-benzodiazepine Z-drugs: zolpidem, zopiclone, zaleplon and benzodiazepines, e.g., diazepam, lorazepam, alprazolam) and “non-GABAergic” (e.g., hydroxyzine, opipramol, quetiapine), while the antidepressants were further categorized as ‘‘SSRI’’ (e.g., fluoxetine, paroxetine, sertraline) and ‘‘non-SSRI’’ (e.g., mirtazapine, trazodone). Psychotropic compounds blocking muscarinic acetylcholine receptors were classified as “anticholinergic” (e.g., opipramol, paroxetine, quetiapine) [18,22]. As a result, some psychotropic medications could fall into more than one category. For example, trazodone, an antidepressant drug with sedative-hypnotic properties, was classified as ‘‘sedative-hypnotic’’ (more specifically: “non-GABAergic sedative hypnotic”) and ‘‘antidepressant’’ (“non-SSRI antidepressant”) and the benzodiazepine, diazepam was classified as “sedative-hypnotic” (“GABAergic sedative-hypnotic”). An SSRI drug, paroxetine was categorized as “antidepressant” (“SSRI antidepressant”) but also “anticholinergic” due to its weak, but clinically significant, antimuscarinic properties [18,22].

### 2.4. Statistics

Sociodemographic and clinical parameters were expressed as the means (±S.D.) or proportions (n/N and %). The severity of OAB and urinary incontinence in subgroups of patients with and without a psychiatric history were compared with the aid of the Student’s *t*-test. The same test was used to compare subgroups of patients treated and untreated with different psychotropic medications (e.g., currently taking vs. non-taking any SSRI).

The one-way analysis of covariance (ANCOVA) was used to control for possible confounders, i.e., age, Body Mass Index (BMI), and the number of medical states other than psychiatric disorders. A probability level (*p*) less than 0.05 was considered significant. The study was exploratory in nature and no correction for multiple comparisons was applied. All statistical analyses were performed using the Statistica 10.0 software package (StatSoft, Tulsa, OK, USA).

## 3. Results

Table 1 shows the basic sociodemographic and clinical characteristics of the study group.

Notably, a significant proportion of patients presented OAB symptoms associated with urinary incontinence (87.4%). A smaller proportion had UUI only (“OAB wet”; 20.5%) and a larger group presented MUI symptoms (both UUI and SUI; 66.9%). The large percentage of women with some SUI symptoms was not surprising as the study group consisted of patients who were generally older, postmenopausal, overweight, and with somatic comorbidities (see Table 1 for details).

### 3.1. Psychiatric History and Severity of OAB and Incontinence Symptoms

Thirty-four of the 127 patients (26.7%) had a lifetime psychiatric history. A psychiatric history was associated with higher IUSS scores, i.e., with more severe OAB symptoms (*t* = 2.22, *p* = 0.02, the Student’s *t*-test; see Table 2 for details). A self-reported history of psychiatric disorder was not associated with the ICIQ-UI-SF, UDI-6, and Stamey test scores (*p*-values > 0.05).

There were no differences in age, BMI, and number of somatic comorbidities between patients with and without a psychiatric history (the Student’s *t*-test, *p*-values > 0.05; data not shown). However, the ANCOVA indicated that BMI [F(1,124) = 5.21, *p* = 0.03], but not age [F(1,124) = 1.35, *p* = 0.24] and number of medical states [F(1,124) = 0.42, *p* = 0.51], was a significant covariate affecting IUSS scores.

### 3.2. History of Depression and Severity of OAB and Incontinence Symptoms

Twenty-six of the 127 patients (20.4%) confirmed a lifetime history of depression. A history of depression was not related to IUSS, ICIQ-UI-SF, and UDI-6 scores (*p*-values > 0.05; Table 2). A history of depression was associated with higher scores on the Stamey test, i.e., more severe SUI symptoms (*t* = 2.3, *p* = 0.02). There were no differences in age, BMI, and the number of somatic comorbidities between the patients with and without a history of depression (*p*-values > 0.05). However, the ANCOVA indicated that BMI [F(1,124) = 11.64, *p* = 0.001], but not age [F(1,124) = 0.30, *p* = 0.58] and number of medical states [F(1,124) = 1.04, *p* = 0.30] was a significant covariate affecting Stamey test scores.

### 3.3. Current Treatment with Psychotropic Medications and Severity of OAB and Incontinence Symptoms

Forty-eight of the 127 patients (37.8%) were treated with at least one psychotropic medication. Taking at least one psychotropic medication was not related to IUSS, ICIQ-UI-SF, UDI-6, and Stamey test scores (*p*-values > 0.05; Table 2).

Nineteen of the 127 patients (15%) were treated with at least two psychotropic medications. These patients had a higher score on the IUSS test (*t* = 2.2, *p* = 0.02). Taking at least two psychotropic medications was not related to ICIQ-UI-SF, UDI-6, and Stamey test scores (*p*-values > 0.05). There were no differences in age, BMI, and the number of somatic comorbidities between the patients treated with at least two psychotropic medications and the other patients (*p*-values > 0.05). However, the ANCOVA indicated that BMI [F(1,124) = 5.62, *p* = 0.02], but not age [F(1,124) = 1.68, *p* = 0.19] and number of medical states [F(1,124) = 0.35, *p* = 0.55] was a significant covariate affecting IUSS scores.

### 3.4. Current Treatment with Antidepressant Medications and Severity of OAB and Incontinence Symptoms

Twenty-four of the 127 patients (18.9%) were treated with antidepressant medication. Taking antidepressant medication was not associated with IUSS, ICIQ-UI-SF, UDI-6, and Stamey test scores (*p*-values > 0.05; Table 2).

Eleven of the 127 patients (8.6%) were treated with an SSRI antidepressant. The patients taking an SSRI had higher Stamey test scores as compared to the patients free of SSRI medication (*t* = 2.1, *p* = 0.04). Taking an SSRI antidepressant was not associated with IUSS, ICIQ-UI-SF, and UDI-6 test scores (*p*-values > 0.05). There were no differences in age, BMI, and the number of somatic comorbidities between the subjects treated with an SSRI and the subjects free of SSRI medication (*p*-values > 0.05). However, the ANCOVA indicated that BMI [F(1,124) = 10.42, *p* = 0.001], but not age [F(1,124) = 0.12, *p* = 0.72] and the number of medical states [F(1,124) = 1.37, *p* = 0.21] was a significant covariate affecting Stamey test scores.

### 3.5. Current Treatment with Sedative-Hypnotics and Severity of OAB and Incontinence Symptoms

Forty of the 127 patients (31.5%) were treated with sedative-hypnotic medication. The subgroups taking and not-taking sedative-hypnotics did not differ in IUSS, ICIQ-UI-SF, UDI-6, and Stamey test scores (*p*-values > 0.05; Table 2).

Twenty-one of the 127 patients (16.5%) were treated with GABAergic sedative-hypnotic medications. Taking GABAergic sedative-hypnotics (benzodiazepines, Z-drugs) was not related to IUSS, ICIQ-UI-SF, UDI-6, and Stamey test scores (*p*-values > 0.05).

Twenty-seven of the 127 patients (21.2%) were treated with sedative-hypnotic medications other than benzodiazepines and Z-drugs. Taking non-GABAergic sedative-hypnotic compounds (e.g., hydroxyzine, opipramol, quetiapine, trazodone) was not related to IUSS, ICIQ-UI-SF, UDI-6, and Stamey test scores (*p*-values > 0.05; Table 2).

### 3.6. Current Treatment with Psychotropic Medications with Anticholinergic Properties and Severity of OAB and Incontinence Symptoms

Thirteen of the 127 patients (10.2%) were treated with at least one psychotropic medication with anticholinergic properties (e.g., opipramol, hydroxyzine, paroxetine, quetiapine). Taking psychotropic drugs with antimuscarinic properties was not related to IUSS, ICIQ-UI-SF, UDI-6, and Stamey test scores (*p*-values > 0.05; Table 2).

## 4. Discussion

To the best of our knowledge, this is the first study showing that a self-reported psychiatric history and current treatment with at least two psychotropic medications may be associated with the severity of OAB in ambulatory urogynecological patients. In addition, our study indicates that in the same group of patients a history of depression and current treatment with SSRI antidepressants may be associated with the severity of SUI symptoms. Treatment with other psychotropic medications, including GABAergic (e.g., benzodiazepines, zolpidem) and non-GABAergic sedative-hypnotics (e.g., hydroxyzine, opipramol, quetiapine, trazodone) was not related to the severity of OAB and incontinence symptoms. Likewise, current treatment with psychotropic drugs with presumed anticholinergic activity was not associated with the severity of OAB and incontinence symptoms. One should remember that our study was cross-sectional in nature and hence it cannot be used to infer causality.

The aim of our study was to reflect a real-life situation when a gynecologist is confronted with OAB patients presenting various histories of psychiatric disorders and taking different psychotropic medications. A routine, detailed assessment of psychiatric disorders may be difficult or even impossible outside the context of the psychiatric care system. One should bear in mind that many individuals tend to deny their psychiatric symptoms due to poor insight. Many also perceive their psychiatric condition as stigmatizing which can make the process of psychiatric interviewing even more difficult [44]. Hence, we assumed that identifying a general psychiatric history was more feasible [26,42] than questioning patients about specific psychiatric diagnoses, such as obsessive-compulsive disorder, panic disorder, or recurrent depressive disorder. We also assumed that obtaining information on psychotropic treatment, including the number of psychotropic medications taken, could provide a simple indicator of psychiatric comorbidities [29,43] in a busy urogynecological clinic.

An increasing body of evidence indicates that psychiatric disorders and OAB symptoms may go together, although causality has not been proven [11,45]. In a prospective, longitudinal study by Perry et al. [10], a large group of women aged 40 years or more, were repeatedly mailed a postal questionnaire including the 30-item Hospital Anxiety and Depression Scale. Cases of de novo urge incontinence were associated with higher levels of anxiety at baseline, but not with depression. Coyne et al. [7] conducted a cross-sectional study based on an Internet survey of men and women aged 40 years or more. Anxiety, but not depression, symptoms were predictive of bothersome (i.e., more severe as described by the patient) OAB symptoms. In a more recent study, Bradley et al. [8] assessed the longitudinal association between selected mental health conditions and OAB in women veterans with the aid of computer-assisted telephone interviews performed at enrollment and one year later. One of the anxiety disorders, but not depression, was predictive of de novo OAB symptoms. In contrast, depression, but not anxiety, was related to OAB remission [8]. Our data further support the latter observations showing that a psychiatric history, but not a specific history of depression, may be associated with the severity of OAB in ambulatory urogynecological patients.

In the present study, a history of depression was associated with the severity of SUI symptoms as measured by the Stamey test. In fact, our patients with SUI symptoms can also be classified as MUI patients with both UUI and SUI components. Our data provide some support for a previous observation in a group of middle-aged women [46] that depression at baseline predicted the incidence of SUI but not UUI. In other studies on subtypes of urinary incontinence and psychiatric comorbidities, the strongest association was found for depression and MUI [47,48]. Taken together, the results of the present and previous studies suggest that patients with MUI symptoms are at a higher risk of depression than patients with other incontinence subtypes.

Treatment with SSRI medications has been associated with more severe SUI symptoms. In this respect, our findings tend to support previous observations on antidepressants and lower urinary tract symptoms. In a cross-sectional, population-based study based on mailed questionnaires, Felde et al. [49] found that the use of antidepressants, but not anxiolytics, was associated with a general risk of urinary incontinence. A study of psychiatric patients [50] undergoing nocturnal polysomnography in a sleep center revealed that treatment with the SSRI drug, sertraline, increased nocturnal urinary frequency as compared to the serotonin-noradrenaline reuptake inhibitor (SNRI), duloxetine. The difference between sertraline and duloxetine could reflect a class effect, with SSRI antidepressants increasing and SNRI antidepressants decreasing nocturnal urinary frequency [50]. Serotonin is thought to be involved in the central and peripheral control of micturition. It has been shown repeatedly that central serotonergic transmission may inhibit micturition in rodents. In contrast, peripheral serotonin, through stimulation of bladder 5-HT_2_, 5-HT_4_, and 5-HT_7_ serotonin receptors, may promote detrusor contraction and facilitate micturition [51,52]. From this perspective, the results of the present and previous studies [49,50] may reflect peripheral rather than central serotonergic control of micturition. It is possible that in some patients treated with SSRI antidepressants increased serotonin levels and stimulation of specific 5-HT receptors in the bladder enhance SUI symptoms.

In the present study, no association was found between the use of SSRI and IUSS, ICIQ-UI-SF, or UDI-6 scores. However, we cannot exclude the possibility that the number of patients treated with SSRIs was too small to detect an association between SSRI treatment and other lower urinary tract symptoms.

The use of sedative-hypnotic medications was not related to the severity of OAB and incontinence symptoms. Several preclinical studies have shown that GABAergic drugs, including benzodiazepines and Z-drugs (e.g., zolpidem), can reduce OAB symptoms through positive modulatory effects on inhibitory GABA_A_ receptors at the peripheral and central level [19,20]. However, it is less clear whether the same control mechanisms exist in human subjects. One should also be aware that the present study was cross-sectional in nature. It is possible that patients taking sedative-hypnotics had more severe baseline OAB and incontinence symptomatology than patients not prescribed sedative-hypnotic medications. In this way, any “positive” drug effect could have been masked by the study protocol, which did not involve longitudinal observations. The same methodological issue may explain the lack of association between the use of psychotropic drugs with anticholinergic activity and OAB/incontinence symptoms.

In the present study, BMI, but not age and number of medical states, was identified as a significant covariate affecting IUSS and Stamey scores. This observation is not surprising as many psychiatric disorders (e.g., depression) and various psychotropic medications are associated with increased BMI, overweight, and obesity [14,18,22]. One may hypothesize that the relationship between urogynecological symptomatology and psychiatric history identified in the present and previous studies [7,8,10] is mediated by an interplay of brain mechanisms and peripheral factors, including body fat accumulation and increased BMI.

The study has several potential limitations, including a relatively small sample size and the fact that all the patients were recruited in a single urban tertiary-care center. Another limitation of the study is its cross-sectional design, which does not allow us to determine any prospective relationship between psychiatric history (and psychotropic medications taken) and urogynecological symptoms. Prospective studies with larger groups of patients are needed to address causality and potential confounders (e.g., age, somatic comorbidities, non-psychotropic medications, socioeconomic status) of the associations described in the present study.

On the other hand, a major strength of the study is that a psychiatric history was evaluated with the simple protocol in outpatients recruited in a naturalistic setting of an ambulatory urogynecological center. In addition, all the patients underwent a urogynecological examination which allowed us to exclude patients with other major comorbidities, such as POP ≥ II and urinary tract infection. In many previous studies on this topic, non-clinical samples were recruited and tested with postal-, telephone-, or internet-based tools [8,10,49]. In fact, in some of these studies, patients with clinically relevant psychiatric disorders were excluded by the study protocol. Another strength of the present study may lie in the fact that qualitative and quantitative data on psychotropic medications taken were gathered. In previous reports on this topic [8,10,45,48], the possible effects of psychotropic treatment were neglected.

In conclusion, our results may provide further support for routine screening for psychiatric comorbidities [8,9,10,11] in urogynecological patients with an overactive bladder.

## Figures and Tables

**Table 1:** Sociodemographic and clinical characteristics of the study group (*n* = 127 patients).

Parameter	
Age (years)	62.9 ± 11.1 ^†^
Body Mass Index (kg/m^2^)	29.4 ± 4.5
Current smokers	19/127 (15%) ^††^
Gravidity	2.1 ± 1.3
Parity	1.8 ± 1.1
Postmenopausal status	110/127 (86.6%)
Number of medical states ^†††^	1.9 ± 1.4
IUSS score	2.2 ± 0.7
ICIQ-UI-SF score	12.3 ± 5.3
UDI-6 score	48.1 ± 18.8
Urinary incontinence	111/127 (87.4%)
Urge urinary incontinence	26/127 (20.4%)
Mixed urinary incontinence ^††††^	85/127 (66.9%)
Stamey test score	1.3 ± 0.9
Psychiatric history	34/127 (26.7%)
History of depression	26/127 (20.4%)
Current treatment with:	
at least one psychotropic medication	48/127 (37.7%)
at least two psychotropic medications	19/127 (14.9%)
any antidepressant	24/127 (18.8%)
any SSRI antidepressant	11/127 (8.6%)
any sedative-hypnotic	40/127 (31.4%)
any GABAergic sedative-hypnotic	21/127 (16.5%)
any non-GABAergic sedative-hypnotic	27/127 (21.2%)
any psychotropic with anticholinergic properties	13/127 (10.2%)

^†^ Mean ± S.D.; ^††^ *n*/N and percentage (%); ^†††^ medical states other than psychiatric disorders (e.g., asthma, hypertension, glaucoma); ^††††^ urge urinary incontinence accompanied by stress urinary incontinence symptoms; IUSS, Indevus Urgency Severity Scale; ICIQ-UI-SF, International Consultation on Incontinence Questionnaire-Urinary Incontinence Short Form; SSRI, selective serotonin reuptake inhibitor; UDI-6, Urinary Distress Inventory-6.

**Table 2:** Psychiatric history, psychotropic medications currently taken and severity of OAB and incontinence symptoms.

Parameter	Subgroups of Patients		*p*-Values
	Psychiatric History (*n* = 34)	No Psychiatric History (*n* = 93)	
IUSS	2.52 ± 0.56 ^†^	2.21 ± 0.74	0.02
ICIQ-UI-SF	13.20 ± 5.19	11.97 ± 5.39	0.25
UDI-6	51.59 ± 18.29	46.86 ± 18.92	0.21
Stamey test	1.61 ± 1.01	1.24 ± 0.91	0.052
	History of depression (*n* = 26)	No history of depression (*n* = 101)	
IUSS	2.50 ± 0.50	2.23 ± 0.75	0.055
ICIQ-UI-SF	13.19 ± 5.32	12.07 ± 5.35	0.34
UDI-6	53.52 ± 18.20	46.74 ± 18.78	0.10
Stamey test	1.73 ± 1.04	1.24 ± 0.91	0.02
	Treatment with at least one psychotropic medication (*n* = 48)	No treatment with psychotropic medications (*n* = 79)	
IUSS	2.45 ± 0.65	2.20 ± 0.74	0.051
ICIQ-UI-SF	12.52 ± 5.69	12.17 ± 5.15	0.73
UDI-6	49.56 ± 18.45	47.25 ± 19.06	0.50
Stamey test	1.45 ± 1.00	1.27 ± 0.91	0.30
	Treatment with at least two (≥2) psychotropicmedications (*n* = 19)	Treatment with ≤1 psychotropic medication (*n* = 108)	
IUSS	2.63 ± 0.59	2.24 ± 0.72	0.02
ICIQ-UI-SF	13.73 ± 6.07	12.05 ± 5.19	0.21
UDI-6	51.75 ± 19.16	47.49 ± 18.75	0.36
Stamey test	1.47 ± 1.12	1.32 ± 0.92	0.53
	Treatment with any antidepressant medication (*n* = 24)	No treatment with antidepressant medications (*n* = 103)	
IUSS	2.54 ± 0.58	2.24 ± 0.73	0.07
ICIQ-UI-SF	13.20 ± 5.34	12.09 ± 5.35	0.36
UDI-6	50.52 ± 18.48	47.57 ± 18.91	0.49
Stamey test	1.62 ± 1.05	1.28 ± 0.92	0.11
	Treatment with any SSRI antidepressant (*n* = 11)	No treatment with SSRI antidepressants (*n* = 116)	
IUSS	2.45 ± 0.68	2.28 ± 0.71	0.45
ICIQ-UI-SF	11.72 ± 4.92	12.36 ± 5.40	0.70
UDI-6	47.72 ± 17.61	48.16 ± 18.98	0.94
Stamey test	1.90 ± 0.83	1.29 ± 0.95	0.04
	Treatment with any sedative-hypnotic medication (*n* = 40)	No treatment with sedative-hypnotic medications (*n* = 87)	
IUSS	2.42 ± 0.63	2.24 ± 0.74	0.18
ICIQ-UI-SF	12.57 ± 5.84	12.18 ± 5.12	0.70
UDI-6	49.68 ± 18.78	47.41 ± 18.87	0.52
Stamey test	1.45 ± 1.01	1.29 ± 0.92	0.41
	Treatment with any GABAergic sedative-hypnotic (*n* = 21)	No treatment with GABAergic sedative-hypnotics (*n* = 106)	
IUSS	2.42 ± 0.67	2.27 ± 0.72	0.36
ICIQ-UI-SF	12.23 ± 6.05	12.32 ± 5.22	0.95
UDI-6	46.42 ± 19.06	48.46 ± 18.82	0.45
Stamey test	1.33 ± 1.01	1.34 ± 0.94	0.65
	Treatment with any non-GABAergic sedative-hypnotic (*n* = 27)	No treatment with non-GABAergic sedative-hypnotics (*n* = 100)	
IUSS	2.44 ± 0.64	2.26 ± 0.73	0.23
ICIQ-UI-SF	12.92 ± 5.86	12.14 ± 5.21	0.50
UDI-6	51.08 ± 17.04	47.33 ± 19.24	0.36
Stamey test	1.48 ± 1.01	1.31 ± 0.93	0.41
	Treatment with any anticholinergic medication (*n* = 13)	No treatment with anticholinergic medications (*n* = 114)	
IUSS	2.30 ± 0.63	2.29 ± 0.72	0.96
ICIQ-UI-SF	10.23 ± 7.16	12.54 ± 5.08	0.14
UDI-6	44.87 ± 17.85	48.50 ± 18.94	0.51
Stamey test	1.23 ± 0.92	1.35 ± 0.96	0.64

^†^ Mean ± S.D.; IUSS, Indevus Urgency Severity Scale; ICIQ-UI-SF, International Consultation on Incontinence Questionnaire-Urinary Incontinence Short Form; SSRI, selective serotonin reuptake inhibitor; UDI-6, Urinary Distress Inventory-6.

## Data Availability

The data presented in this study are available on request from corresponding author.
